# Potential Use of Alpha-1 Anti-trypsin in the Covid-19 Treatment

**DOI:** 10.3389/fcell.2020.577528

**Published:** 2020-10-23

**Authors:** Fernanda Martini, Monica De Mattei, Carlo Contini, Mauro G. Tognon

**Affiliations:** ^1^Laboraotories of Cell Biology and Molecular Genetics, University of Ferrara, Ferrara, Italy; ^2^Department of Medical Sciences, University of Ferrara, Ferrara, Italy

**Keywords:** SARS-CoV-2, COVID-19, AAT activity, cytokine, inflammation, therapy

## Introduction

The coronavirus disease-2019 (COVID-19), firstly originated in the city of Wuhan, Hubei Province, People's Republic of China, is due to infection by the severe acute respiratory syndrome coronavirus 2 (SARS-CoV-2) (Guo et al., 2020). SARS-CoV-2 has shown high infectivity, and high mortality associated to respiratory failure from acute respiratory distress syndrome (ARDS), becoming rapidly a worldwide health emergency (Guo et al., 2020). In spite of the several efforts of researchers, the limited knowledge on the disease progression and immunological profile, and the absence of medications or vaccines proven to be effective for treatment or prevention of the SARS-CoV-2, lead to the urgent need for efficient and safe therapies, and treatments to limit acute inflammation associated with severe pneumonia and mortality (Mirastschijski et al., 2020). Agents such as potent anti-inflammatory drugs, some antivirals including Remdesevir and, recently, hyperimmune plasma, seem promising, whereas several studies are currently ongoing to test and prove their effectiveness (Guo et al., 2020).

Indeed, approved safe therapies with potential ability to control infection and to prevent respiratory complications may be preferential candidates (Zhou et al., 2020), while the range of proposal drugs is rapidly growing (Scalise and Indiveri, 2020; Ye et al., 2020). This approach may readily permit to identify and use safe drugs, until knowledge on the viral biology will allow to identify specific SARS-CoV-2 drugs and/or vaccines.

## Alpha-1 Antitrypsin (AAT)

Alpha-1 antitrypsin (AAT) protein is one of the major serum proteins involved in anti-inflammatory processes (de Serres and Blanco, 2014). AAT, mainly synthesized in the liver, is released into the bloodstream. AAT is primarily known as a serine protease inhibitor (SERPIN) targeting several enzymes involved in tissue damage/repair, whereas its activity is very high in the lower respiratory tract where it provides over 90% of the defenses against proteases, mainly neutrophil elastase (NE), protecting healthy tissues from the digestive action of proteolytic enzymes. As an acute phase protein, AAT increases 4-6 fold during infections, inflammation, tissue injury, surgery and late pregnancy (Sanford et al., 1999; Buttenschoen et al., 2001; Ziakas et al., 2011; de Serres and Blanco, 2014).

Further, AAT displays immunomodulatory abilities. It inhibits pro-inflammatory cytokines, while increasing anti-inflammatory mediators (Guttman et al., 2015). Specifically, AAT functions have been linked to interleukin 6 (IL6) signaling (Yuan et al., 2018). Also, AAT has shown anti-viral activities in *in vitro* and *in vivo* studies (Shapiro et al., 2001; Munch et al., 2007; Wanner et al., 2012). In primary Rhesus monkey kidney cells, AAT inhibited H1N1 influenza virus cell infection, whereas in infected mice AAT decreased the mortality rates and inflammatory cytokines (Wanner et al., 2012). In HIV infection, AAT suppressed viral production in chronically infected monocytes, inhibited HIV-1 entry into a cell line designed to detect viral entry, and reduced HIV-1 replication in human peripheral mononuclear cells (Shapiro et al., 2001; Munch et al., 2007).

AAT is widely studied in clinical setting due to the existence of congenital genetic defects that reduce its concentrations in the blood (Bornhorst et al., 2007). More than 100 allelic variants of the gene coding for the AAT, called Serine Protease Inhibitor-A1 (*SERPINA1*) exist, whereas many of them are associated with reduced circulating protein levels or altered protein activity (Bornhorst et al., 2007). Individuals with AAT deficiency (AATD), in the presence or in the absence of concomitant factors such as smoke, environmental pollutants, and age, have a very high risk of developing pathologies of the respiratory system, such as pulmonary emphysema and chronic obstructive pulmonary disease (COPD) associated to a progressive damage of lung parenchyma (Nuñez et al., 2020).

Additionally, reduced levels of AAT or abnormal AAT proteins have been associated with increased susceptibility to viral infections, such as hepatitis B, hepatitis C, HIV-1, and HTLV-1 infections (Hashemi et al., 2005; Settin et al., 2006; da Silva Ferreira et al., 2014, 2017), and the development of autoimmune and chronic inflammatory diseases, such as diabetes mellitus and panniculitis (Hashemi et al., 2006; de Serres and Blanco, 2014).

## Alpha-1 Antitrypsin Therapy in COPD

Currently, AAT therapy, which is a FDA approved drug, is the only available pharmacological treatment that can slow COPD progression in AATD patients (Griese and Scheuch, 2016; Brantly et al., 2018). COPD is a respiratory disease characterized by persistent respiratory symptoms with significant obstruction of airflow, and increased lung and systemic inflammation (Bradford et al., 2017; Celli and Wedzicha, 2019).

The progression of COPD is associated with increased inflammation of the airway and alveolar wall (Chen et al., 2016). Patients affected by COPD frequently present exacerbations, often triggered by bacterial/viral respiratory infections or viruses, which lead to disease progression through an exaggerated inflammatory response, therefore requiring pharmacological treatments (Xiong et al., 2017). COPD exacerbations are characterized by high neutrophil counts, and high levels of C-reactive protein (CRP), as well as inflammatory cytokines including tumor necrosis factor α (TNFα), interleukin-1 (IL1), interleukin-8 (IL8) and IL6 (Bradford et al., 2017; Xiong et al., 2017; Nuñez et al., 2020).

Intravenous AAT therapy has been used for the treatment of individuals with AATD and COPD since the late 1980s. Clinical AAT efficacy has been reported showing therapeutic effect on FEV1 and CT lung densitometry in observational or registry studies (Chapman et al., 2009, 2015). Further, AAT therapy impacts on several biological parameters involved in COPD and its progression in AATD patients (Campos et al., 2019). AAT therapy restores the serum protein levels to those of normal subjects, significantly reduces protease activities and inflammation by downregulating several inflammatory markers (Brantly et al., 2018; Campos et al., 2019).

The AAT multiple activities have suggested a potential therapeutic use of AAT for the treatment of several inflammatory and autoimmune diseases beyond COPD, as well as viral diseases including HIV-1 infection, whereby AAT infusion can decrease HIV viral load (Forssmann et al., 2010; Lewis, 2012; Wanner et al., 2012). Therefore, the research on the possible benefits of AAT therapy in other diseases is ongoing, particularly in transplant and type-1 diabetes, as well as in rheumatologic diseases (Lewis, 2012; Marcondes et al., 2016). In preclinical studies, beneficial effects of AAT treatment have been observed in autoimmune disease models, such as rheumatoid arthritis and systemic lupus erythematosus, whereas some clinical studies have investigated AAT treatment in graft vs. host disease (GVHD) in organ transplantation and type 1 diabetes mellitus (Grimstein et al., 2011; Lewis, 2012; Wanner et al., 2012; Marcondes et al., 2016).

## Discussion

In COVID-19 patients, disease severity and high mortality are associated to respiratory failure from ARDS and multiple organ dysfunctions due to an impressive cytokine storm, with significant increased levels of several inflammatory mediators, including IL-6, interferon gamma (INFγ), TNFα, interleukin 17 (IL-17), IL-8 (McElvaney et al., 2020; Pedersen and Ho, 2020). Further, the neutrophil-to-lymphocyte ratio (NLR) in peripheral blood, considered a systemic inflammatory biomarker, is increased in patients with COVID-19 with severe disease compared to those with mild disease and healthy controls (McElvaney et al., 2020). Because elevated levels of IL-6 have been associated to poor prognosis and predictor of mortality, the use of IL6 antagonists has been early proposed for the COVID-19 treatment. Recently, the treatment with the monoclonal antibody Tocilizumab, which is currently used for the treatment of rheumatoid arthritis, has been considered an attractive approach for the treatment of COVID-19 (Guaraldi et al., 2020). Clinical trials to study the efficacy and safety of the Tocilizumab monoclonal antibody are ongoing in several Countries, including Italy (Guaraldi et al., 2020). Nevertheless, the validity of the treatment is debated (Arnaldez et al., 2020). In fact, IL-6 is a crucial inflammatory cytokine for the development of antibodies and the activation of T lymphocytes against infectious agents, and its inhibition could even be deleterious, since it would lower the immune response against the SARS-CoV- 2 (Arnaldez et al., 2020).

To date AAT supplement therapy is largely used to avoid disease exacerbation in AATD COPD patients (Brantly et al., 2018; Campos et al., 2019). In spite of several clinical differences, COVID19 and COPD patients may present similar clinical outcome, such as acute exacerbations, resulting in exaggerated inflammatory response and increased NRL and IL6 levels (Chen et al., 2016; Wang et al., 2020). Interestingly, among common comorbidities in COVID19, Wang et al. have shown that COPD is associated with a 5.9-fold higher risk of progression in patients with COVID-19 (Wang et al., 2020). One explanation could be that the expression of angiotensin-converting enzyme 2 (ACE2), which is the host cell receptor for SARS-CoV-2 entry, is increased in COPD patients (Leung et al., 2020).

The potential clinical utility of AAT treatment in COVID-19 patients is based on the following considerations: (i) AAT acts as an efficient inhibitor of the host transmembrane protease serine 2 (TMPRSS2) protein receptor (Azouz et al., 2020), which is essential during the initial phase of the SARS-CoV-2 infection. Indeed, similar to other coronaviruses, SARS-CoV-2 binds its envelope spike (S) protein ligand to the ACE2 receptor for entering into cells (Azouz et al., 2020). The host TMPRSS2 receptor acts by processing the viral S protein, allowing S protein–ACE2 interaction and infection of the host cell. Consequently, the AAT treatment may limit the SARS-CoV-2 entry into host cells, which represents the first step of the viral infection; (ii) despite AAT increase in COVID-19 patients, according to its role as an inflammatory acute phase protein, it has been shown that the IL6:AAT ratio is higher in patients with the severe/critical disease than in mild/stable disease (McElvaney et al., 2020). Moreover, in severe/critical COVID-19 patients the increase and reduction of the IL-6:AAT ratio has been associated with a poor outcome and clinical improvement, respectively (McElvaney et al., 2020). Therefore, even though AAT levels are correctly risen in COVID-19 patients, AAT augmentation may be useful to modulate the production and activity of key pro-inflammatory cytokines, while preserving the production of the anti-inflammatory cytokine IL-10 (Guttman et al., 2015); (iii) AATD is a largely under-recognized condition (De Serres et al., 2003). AATD state by itself is not a disease, but a genetic background/predisposition to the development of several diseases. One could speculate that individuals carrying *SERPINA1* deficient allelic variants may be at higher risk of SARS-CoV-2 infection than those with wild type *SERPINA1*, as previously shown for retroviral infections (Hashemi et al., 2005; Settin et al., 2006; da Silva Ferreira et al., 2014, 2017). It is also possible to hypothesize that some *SERPINA1* deficient allelic variants could not allow AAT to be sufficiently increased or efficient to counteract SARS-CoV-2 infection, leading to the progressive worsening of the COVID-19 disease. Of note, the Italian register of the AATD reports a higher frequency of cases in northern Italy than in central and southern Italy, with the highest incidence in Lombardy (47%), which is the northern Italian region counting the highest mortality rate for COVID-19, reaching up 85% cases (Luisetti et al., 2015; World Health Organization., 2020). This possible association between AATD and COVID-19 deserves to be explored, and might reveal subtypes of COVID-19 patients who could benefit from AAT supplementary therapy. In this view, AAT treatment should be also useful to prevent severe outcome in AATD COVID-19 patients presenting mild symptoms.

Based on these observations, it is reasonable to suggest that AAT treatment, used to slow COPD progression, may be considered in COVID-19 patients. As AAT administration is a FDA-approved drug with a confirmed safety profile, this novel therapeutic potential makes AAT a promising candidate to counteract COVID-19 disease. The use of AAT in the treatment of COVID-19 patients will allow to overcome the limits of the Tocilizumab therapy, which could limit the immune response against SARS-CoV-2 (Arnaldez et al., 2020). Furthermore, while treatment with Tocilizumab is limited to blocking only one pro-inflammatory pathway (IL6/IL6-R), the administration of AAT could have a broader immunomodulatory and “pro-resolving” effect, increasing the chances of clinical success. In fact, from a biochemical point of view, AAT acts both extracellularly as a protease inhibitor and as a ligand of some membrane receptors, inducing a signal, which modulates the immune response (Guttman et al., 2015). AAT is able: (i) to polarize macrophage cells toward a M2 phenotype, known to be anti-inflammatory and promoter of tissue repair/regeneration programs (Guttman et al., 2016). This activity is significant, since monocyte-macrophages are the main players in the initiation and maintenance of the inflammatory cascade induced by SARS-CoV-2 (Guo et al., 2020); (ii) to neutralize the elastase released by neutrophil granulocytes, also involved in the pathogenesis of lung damage; (iii) to favor the differentiation of T lymphocytes toward the Treg phenotype, endowed with immunosuppressive properties that could contribute the shutdown of the cytokine storm (Baranovski et al., 2015).

Finally, engineered AAT (α1-PDX) converted to a furin inhibitor may be a promising antiviral molecule for SARS-CoV-2 (Scott and Sheffield, 2020). The human serine proteinase furin is a proprotein convertase, which processes different pathogens for entering host cell (Shiryaev et al., 2007; Coutarda et al., 2020). Furin-mediated cleavage of viral glycoproteins (gp) facilitates HIV-1, measles, and influenza viral infections (Thomas, 2002). α1-PDX can block HIV-1 gp160 processing, which is required for cellular invasion, whereas it reduces HIV-1 and measles infectivity *in vitro*. On this ground, α1-PDX can be considered as a potential treatment strategy for COVID-19 (Anderson et al., 1993; Watanabe et al., 1995; Bahbouhi et al., 2000).

In conclusion, due to the known roles of AAT and its current use in clinics (Chapman et al., 2009, 2015; Forssmann et al., 2010; Lewis, 2012; Griese and Scheuch, 2016; Marcondes et al., 2016; Brantly et al., 2018; Campos et al., 2019), AAT treatment may represent a safe drug with potential activity in treating COVID-19 affected patients. Safe protocols for treatments, including doses and timely infusions are currently available for COPD patients (Chapman et al., 2009, 2015; Griese and Scheuch, 2016; Brantly et al., 2018; Campos et al., 2019). AAT treatment via aerosol, used in COPD treatment, could be administered in COVID-19 patients as well (Griese and Scheuch, 2016). AAT local instillation or aerosol in humid heat vaporization (40–41°C) in the first phase of COVID-19 might be a powerful strategy for hampering SARS-CoV-2 entering into the host cells by inhibiting host cell receptors, significantly decreasing viral replication, risk of evolution to the more severe clinical pictures, reducing hospitalization and death rate. As recently proposed for Remdesivir, local aerosolisation of AAT with hot-wet humid water vaporization (WHV) at 40–41°C could significantly decrease viral replication in hours or 2–3 days at most, in the early stages of respiratory disease (Contini et al., 2020). The amount of product required may be minimal, plausibly reducing disease evolution, patient pain and discomfort, the adverse effects of intravenous administration and social cost.

We suggest that AAT treatment deserves the attention of clinicians for its potential utility in the treatment of COVID-19. Further studies evaluating the levels and the activity of AAT in COVID-19 patients presenting cytokine storm or affected by AATD will lead to stratification of patients, assessing whether AAT treatment may be useful as a therapy or to prevent severe outcomes.

## Author Contributions

FM proposed the hypothesis. MD wrote the first draft. FM, CC, and MT corrected the draft. FM, MD, CC, and MT wrote the final text. MT supervised the work and submitted the manuscript.

## Conflict of Interest

The authors declare that the research was conducted in the absence of any commercial or financial relationships that could be construed as a potential conflict of interest.
